# Glycerol-3-Phosphate Acyltransferase GPAT9 Enhanced Seed Oil Accumulation and Eukaryotic Galactolipid Synthesis in *Brassica napus*

**DOI:** 10.3390/ijms242216111

**Published:** 2023-11-09

**Authors:** Wei Gong, Wenling Chen, Qiang Gao, Lei Qian, Xueyuan Yuan, Shaohua Tang, Yueyun Hong

**Affiliations:** National Key Laboratory of Crop Genetic Improvement, Huazhong Agricultural University, Wuhan 430070, China; gwei@webmail.hzau.edu.cn (W.G.); chenwl828@163.com (W.C.); gaoq@webmail.hzau.edu.cn (Q.G.); 15997130414@163.com (L.Q.); yuanxueyuan@webmail.hzau.edu.cn (X.Y.); amy.feather@163.com (S.T.)

**Keywords:** glycerol-3-phosphate acyltransferase, phosphatidic acid, oil accumulation, eukaryotic glycerolipids, *Brassica napus*

## Abstract

Glycerol-3-phosphate acyltransferase GPAT9 catalyzes the first acylation of glycerol-3-phosphate (G3P), a committed step of glycerolipid synthesis in *Arabidopsis*. The role of *GPAT9* in *Brassica napus* remains to be elucidated. Here, we identified four orthologs of GPAT9 and found that BnaGPAT9 encoded by BnaC01T0014600WE is a predominant isoform and promotes seed oil accumulation and eukaryotic galactolipid synthesis in *Brassica napus*. *BnaGPAT9* is highly expressed in developing seeds and is localized in the endoplasmic reticulum (ER). Ectopic expression of BnaGPAT9 in *E. coli* and siliques of *Brassica napus* enhanced phosphatidic acid (PA) production. Overexpression of *BnaGPAT9* enhanced seed oil accumulation resulting from increased 18:2-fatty acid. Lipid profiling in developing seeds showed that overexpression of *BnaGPAT9* led to decreased phosphatidylcholine (PC) and a corresponding increase in phosphatidylethanolamine (PE), implying that BnaGPAT9 promotes PC flux to storage triacylglycerol (TAG). Furthermore, overexpression of *BnaGPAT9* also enhanced eukaryotic galactolipids including monogalactosyldiacylglycerol (MGDG) and digalactosyldiacylglycerol (DGDG), with increased 36:6-MGDG and 36:6-DGDG, and decreased 34:6-MGDG in developing seeds. Collectively, these results suggest that ER-localized BnaGPAT9 promotes PA production, thereby enhancing seed oil accumulation and eukaryotic galactolipid biosynthesis in *Brassica napus*.

## 1. Introduction

Vegetable oils are essential nutrients and high-density energy compounds of food and animal feed, and also serve as an important feedstock for the chemical industry and a renewable substitute for biodiesel [1,2]. *Brassica napus* (AACC, 2n = 38), an allopolyploid generated from natural hybridization of *Brassica rapa* (AA, 2n = 20) and *Brassica oleracea* (CC, 2n = 18) [3,4], is an oil crop grown worldwide that contributes to approximately 16% of vegetable oil for human consumption globally [5]. Vegetable oil, in the form of triacylglycerol (TAG), is mainly accumulated in seeds of *Brassica napus*, which is biosynthesized from fatty acid (FA) esterified at glycerol backbone. The seed oil of *Brassica napus* predominantly consists of oleic acid (C18:1, >60%), and is rich in linoleic acid (C18:2, ~22%) and α-linolenic acid (C18:3, ~10%), which is beneficial for human health [5]. With an increasing population and limited arable land, the improvement of oil content is emerging as a critical goal of oil crop breeding [1,6].

Glycerolipids such as TAG, phospholipids, and galactolipids are derived from phosphatidic acid (PA), an important intermediate in lipid metabolism [7,8]. In plants, PA is de novo synthesized by stepwise acylation at the sn-1 and sn-2 positions of glycerol-3-phosphate (G3P) by glycerol-3-phosphate acyltransferase (GPAT) and lysophosphatidic acid acyltransferase (LPAT), respectively, which occurs in two pathways, the prokaryotic pathway in plastids (chloroplasts) and eukaryotic pathway in the endoplasmic reticulum (ER) [8,9]. In the prokaryotic pathway, the plastid-localized GPAT (also known as ATS1) catalyzes the sn-1 acylation of G3P to produce lysophosphatidic acid (LPA), which is followed by the sn-2 acylation of LPA by plastidial LPAT (ATS2) to produce PA using acyl-acyl carrier protein (acyl-ACP) as an acyl donor [10,11,12]. PA derived from the prokaryotic pathway is used for phosphatidylglycerol (PG) synthesis, while diacylglycerol (DAG) derived from PA dephosphorylation serves as a precursor for galactolipids such as monogalactosyldiacylglycerol (MGDG) and digalactosyldiacylglycerol (DGDG), the major components of photosynthetic membranes [8,9,13]. In the eukaryotic pathway, PA is generated by the sn-1 and sn-2 acylations of G3P catalyzed by ER-localized GPAT and LPAT, respectively, using acyl-CoA as an acyl donor [8,9,14,15,16]. The eukaryotic PA and its dephosphorylated DAG provide precursors for TAG and phospholipids such as PG, phosphatidylcholine (PC), phosphatidylserine (PS), phosphatidylethanolamine (PE), and phosphatidylinositol (PI) [8,9]. Alternatively, the eukaryotic-derived glycerolipids can be reentered into the chloroplasts, presumably in the form of PA or DAG for the synthesis of photothylakoid membrane lipids [17,18,19,20]. Thus, the eukaryotic glycerolipids initiated by ER-localized GPAT activity may affect lipid metabolism within both the ER and the plastid.

TAG synthesis in plants is more complicated than in animals, and plants synthesize TAG via two main routes with distinct acyl donor at the sn-3 position of TAG. One route is the acyl-CoA-dependent known as the Kennedy pathway through three sequential acylations at the sn-1, sn-2, and sn-3 positions of G3P backbone, catalyzed by GPAT, LPAT, and diacylglycerol acyltransferases (DGAT), respectively, using acyl-CoA as an acyl donor to generate TAG [21,22,23]. Another route is the acyl-CoA-independent, in which phospholipid: diacylglycerol acyltransferase (PDAT) transfers an acyl group of PC to the sn-3-position of DAG moiety to produce TAG [24,25]. In addition, the fatty acid species at sn-1 and sn-2 positions of TAG can be derived from PC through exchange between DAG and PC catalyzed by phosphatidylcholine: diacylglycerol cholinephosphotransferase (PDCT) [26].

GPAT catalyzes the first acylation at the backbone of glycerolipids, which is involved in the synthesis of membrane lipids, storage lipids, and extracellular lipids using acyl-CoA, acyl-ACP, or fatty-acid derivative (ω-hydroxy and α,ω-dicarboxylic FAs) [8]. The GPAT family contains multiple members and has been identified in various plant species, such as *Arabidopsis*, rapeseed, rice, maize, and tomato [12,15,27,28,29,30,31,32]. *Arabidopsis* contains 10 GAPTs, which are localized to plastid, ER, and mitochondria [8]. The plastid-localized GPAT (ATS1) is a soluble protein catalyzing the sn-1 acylation of G3P to produce sn-1 acyl-LPA involved in the synthesis of prokaryotic membrane lipids using acyl-ACP as substrate [12,33]. Loss of ATS1 leads to growth retardation with a marked reduction in prokaryotic glycerolipids such as PG and MGDG in *Arabidopsis* [10,12]. GPAT1 to GPAT8 are membrane-associated and land plant-specific GPATs that catalyze the acylation at the sn-2 position of G3P and prefer unusual FAs (ω-oxidative FAs) to produce sn-2 acyl-LPA for the synthesis of extracellular lipids such as cutin and suberin [27,28,30,34,35,36]. *Arabidopsis* AtGPAT1-3 is mitochondria-localized, and AtGPAT1 is required for cutin synthesis and normal microspore development [27]. AtGAPT4-8 is the ER-localized GPATs, in which AtGAPT4, 6, 8 have dual activities with the sn-2 acyltransferase and phosphatase toward G3P to produce 2-monoacylglycerol (2-MAG) for cutin synthesis [34,35,36], while AtGPAT5 and AtGPAT7 are vascular higher plant-specific GPATs with sn-2 acylation activity involved in suberin synthesis [28,30]. Therefore, GAPT1–GPAT8 are involved in the synthesis of extracellular lipids rather than membrane lipids and storage TAG.

Recent studies have shown that AtGPAT9 is the only one involved in the eukaryotic intracellular lipids such as membrane lipids and storage TAG in *Arabidopsis* [15,16]. Complete loss of GPAT9 resulted in the lethality of male and female gametophytes in *Arabidopsis* [15]. Suppressed GPAT9 led to reduced contents of seed oil and total polar lipids [15,16]. However, the gene encoding the ER-localized GPAT responsible for oil synthesis is unknown in *Brassica napus*. Moreover, whether GPAT9 affects individual phospholipid and galactolipid remains to be characterized in plants. One ortholog of AtGPAT9, BnaC01T0014600WE, hereafter named BnaGPAT9, is highly expressed in various tissues with highest in developing seed, implying its role in seed oil accumulation. Here, we functionally characterized the involvement of BnaGPAT9 in seed oil synthesis and found that overexpression of *BnaGPAT9* enhanced seed oil accumulation and eukaryotic galactolipid biosynthesis in *Brassica napus*.

## 2. Results

### 2.1. Identification of an Ortholog of AtGPAT9 in Brassica napus

**Figure 1:**
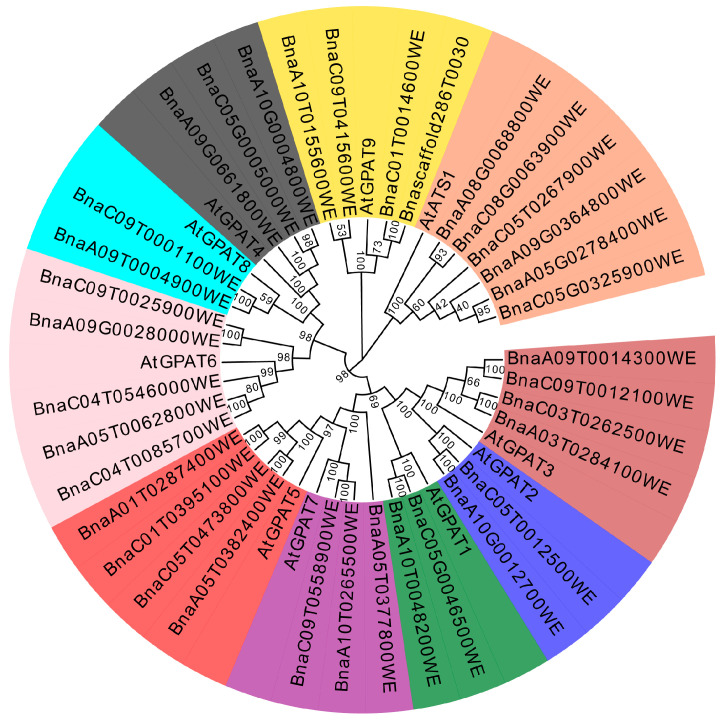
Phylogenetic analysis of GPAT homologues from different plant species. The phylogenetic analysis was constructed using Mega 11 software (version 11.0.13), with the Neighbor-Joining method, Poisson model, and 1000 Bootstrap test. The phylogenetic analysis was embellished by EvolView. At, *Arabidopsis thaliana*; Bna, *Brassica napus*.

To identify genes encoding GPAT in *Brassica napus*, we first applied BLAST to search the databases (https://www.genoscope.cns.fr/brassicanapus/, accessed on 20 November 2018), (http://cbi.hzau.edu.cn/bnapus/index.php, accessed on 20 November 2018), and (https://www.ncbi.nlm.nih.gov/, accessed on 20 November 2018) by querying *Arabidopsis* GPAT sequences (https://www.arabidopsis.org, accessed on 20 November 2018), and using keywords of glycerol-3-phosphate acyltransferase. A total of 35 GPAT genes were obtained from *Brassica napus* genome (Figure 1). Phylogenetic relationships showed that all GPATs from *Brassica napus* together with their respective orthologs in *Arabidopsis* were classified into two types, type I and type II. Type I, involved in the prokaryotic pathway, includes *Arabidopsis* plastid-localized ATS1 and its six orthologs in *Brassica napus*. Type II, involved in the eukaryotic pathway, includes *Arabidopsis* AtGPAT1-9 and their counterparts in *Brassica napus*, in which AtGPAT9 and its orthologs are more distantly related to GPAT1-8 members (Figure 1). GAPT1-8 can be further divided into three groups including group I with GPAT1, GPAT2, and GPAT3, group II with GPAT4, GPAT6, and GPAT8, and group III with GPAT5 and GPAT7, based on phylogenetic relationship (Figure 1).

*Brassica napus* genome contains four *BnaGPAT9* homologs (BnaC01T0014600WE, BnaA10T0155600WE, BnaC09T0415600WE, Bnascaffold286T0030000WE), sharing 94.15%, 92.55%, 92.55%, and 94.15%, respectively, in deduced amino acid sequence similarity to *Arabidopsis* AtGPAT9. Sequence comparison and structural domain analysis of the amino acid sequences of four BnaGPAT9s and AtGPAT9 revealed that they possess three conserved transmembrane domains (TM I–TM III) and a highly conserved acyltransferase catalytic domain with the four conserved motifs (Block I–Block IV, Supplementary Figure S1).

### 2.2. BnaGPAT9 Is Highly Expressed in Developing Seed and BnaGPAT9 Is Localized to ER

**Figure 2:**
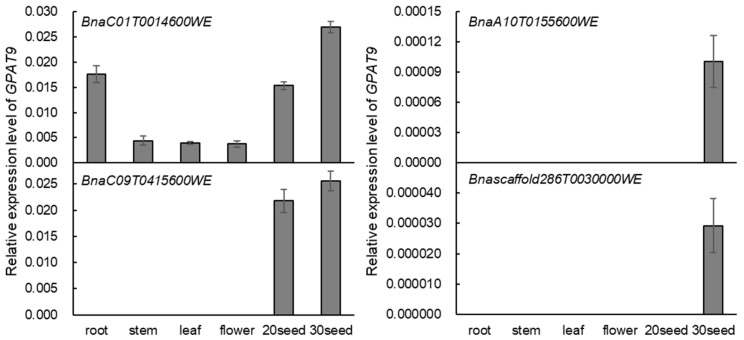
*BnaGPAT9* expression pattern in various tissues in *Brassica napus* by quantitative RT-PCR. The roots, stems, and leaves were sampled from 50-day-old plants at the vegetative stages, whereas the flowers and seeds were sampled from the plants at the flowering and seed setting stages. The seed samples were collected from seeds 20 and 30 days after pollination. *BnaActin* (BnaC02T0031300WE) was used as an internal standard control. Values are means ± SD (*n* = 3).

**Figure 3:**
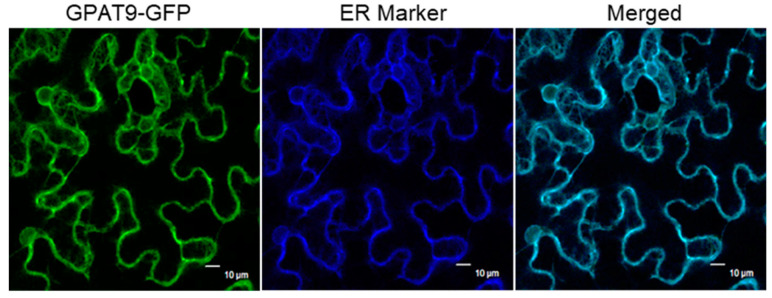
BnaGPAT9 is localized to the ER. BnaGPAT9 fused with green fluorescent protein (GFP) at C-terminus was transiently co-expressed with ER marker protein-CFP (CD3-953) in the epidermal cells of *N*. *benthamiana* leaves. The fluorescent images were observed using a confocal laser scanning microscope. Scale bar = 10 μm.

To investigate the temporal and spatial distribution of *BnaGPAT9* mRNA in *Brassica napus*, the expression levels of four homologous *BnaGPAT9s* in various tissues at the vegetative and reproductive stages were analyzed by quantitative reverse-transcription PCR (RT-qPCR). The expression patterns of four *BnaGPAT9*s in *Brassica napus* were substantially different, among which *BnaC01GPAT9* (BnaC01T0014600WE), located on chromosome 1 of the C genome, was highly expressed in all tissues, including root, stem, leaf, flower, and developing seed, with highest in root and developing seeds 20 and 30 days after pollination (DAP). The expression level of *BnaC09GPAT9* (BnaC09T0415600WE) was negligible in most tissues such as root, stem, leaf, and flower, but it is high in developing seeds 20 and 30 DAP (Figure 2). By contrast, the transcripts of *BnaA10GPAT9* (BnaA10T0155600W) and *BnascaGPAT9* (Bnascaffold286T0030000WE) were barely detected in various tissues tested (Figure 2). These results showed that *BnaC01GPAT9* (BnaC01T0014600WE) is predominant and abundant in various tissues, particularly in developing seeds, implying its roles in seed oil synthesis and plant growth. Therefore, we chose *BnaC01GPAT9* (BnaC01T0014600WE, referred to herein as BnaGPAT9) for further characterization in this study. To investigate the subcellular localization, BnaGPAT9 was fused with GFP at its C-terminus and transiently expressed in leaf epidemic cells of tobacco. The green fluorescent signal of BnaGPAT9-GFP detected and was overlaid with blue fluorescent signal of the ER marker protein CD3-CFP (Figure 3), suggesting that BnaGPAT9 is localized to the ER. These results imply that BnaGPAT9 may play a role in eukaryotic glycerolipid assembly in *Brassica napus*.

### 2.3. Overexpression of BnaGPAT9 Enhancing Oil Accumulation in Seeds

**Figure 4:**
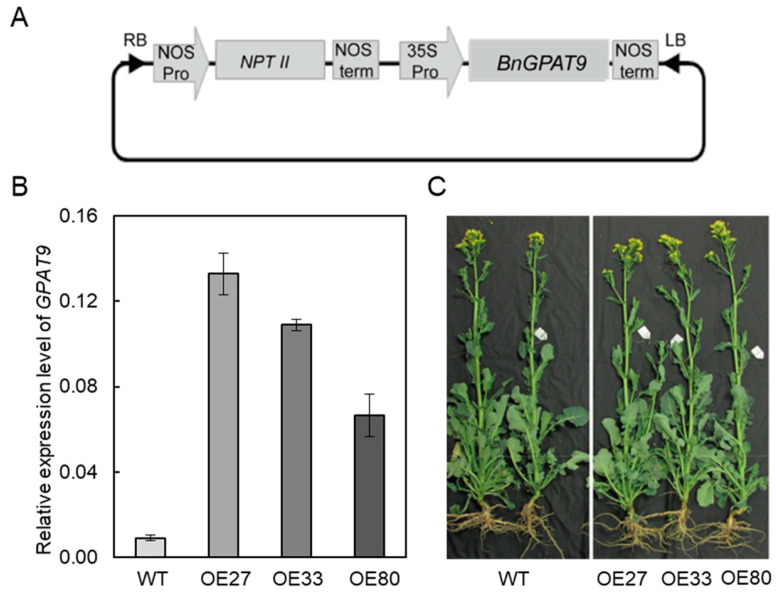
Overexpression of *BnaGPAT9* in *Brassica napus.* (**A**) The construct containing *BnaGPAT9* in the binary vector pBI121. (**B**) RT-qPCR measurements of *BnaGPAT9* transcript level in wild-type (WT), *BnaGPAT9*-OE27, OE33, and OE80 plants. Total RNA was extracted from the leaves of 50-day-old plants. The relative expression level of *BnaGPAT9* was normalized to *BnaActin.* Values are means ± SD (*n* = 3). (**C**) The growth phenotypes of five-month-old plants grown in the field.

The highest expression level of *BnaGPAT9* in developing seeds suggests that *BnaGPAT9* may be involved in oil synthesis in seeds. To test this hypothesis, the full length of the coding sequence of *BnaGPAT9* was amplified from mRNA extracted from leaves of cultivar Westar (*Brassica napus*) and overexpressed in Westar plants under the control of 35S promoter (Figure 4A). Three independent *BnaGPAT9*-overexpression (OE) lines, OE27, OE33, and OE80, were used for further studies. The *BnaGPAT9* average expression level in *BnaGPAT9*-OE plants was a seven-fold increase as compared to wild-type (WT) plants (Figure 4B). The growth and development were comparable between *BnaGPAT9*-OE and WT plants under the field growth conditions (Figure 4C).

**Figure 5:**
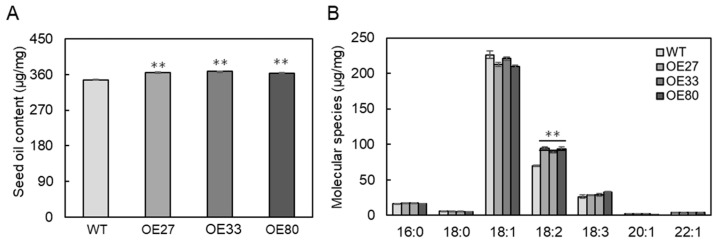
Overexpression of *BnaGPAT9* enhanced seed oil accumulation. Total oil content (**A**) and fatty acid composition (**B**) in mature seeds. Values are means ± SD (*n* = 3). ** denotes significance at *p* < 0.01 compared with wild type (WT) based on Student’s *t*-test.

To investigate the effect of BnaGPAT9 on seed oil accumulation, the mature seeds were collected for oil analysis. The seed oil content of *BnaGPAT9*-OE was significantly higher than that of WT. The seed oil contents in OE27, OE33, and OE80 lines were increased by 5.1%, 6.1%, and 4.8%, respectively, as compared to WT (Figure 5A). Moreover, *BnaGPAT9*-OE also affected the oil composition, with increased linoleic acid (18:2) accompanied by slightly decreased oleic acid (18:1) compared to WT (Figure 5B). The results suggest that BnaGPAT9 promotes seed oil accumulation through enhanced 18:2 fatty acid.

### 2.4. BnaGPAT9 Promoted PA Production In Vitro and In Vivo

**Figure 6:**
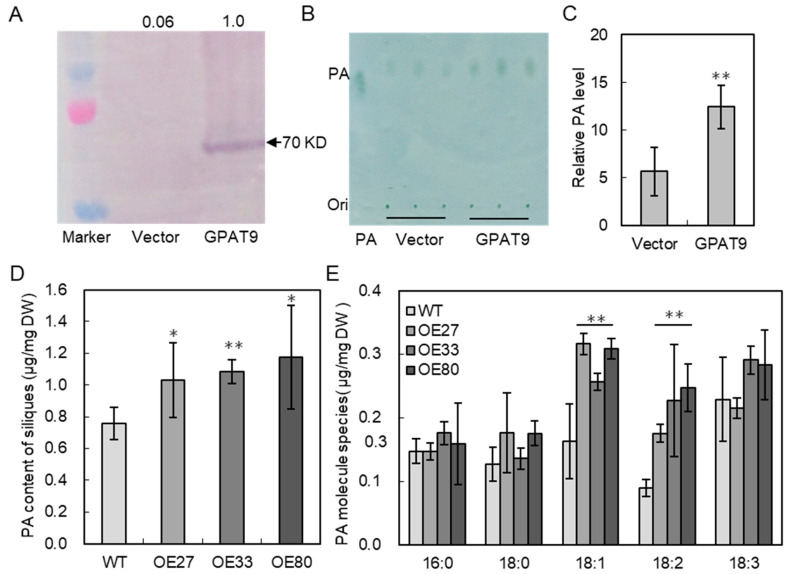
BnaGPAT9 enhanced PA production in vitro and in vivo. (**A**) Immunoblot of His-tagged BnaGPAT9 produced in *E. coli* BL21 (DE3) cells using anti-His antibody. (**B**,**C**) The enzyme activity of BnaGPAT9 using C18:1-CoA as an acyl donor. PA in thin-layer chromatography (TLC) was visualized by bromothymol blue (**B**) and the relative amount of PA was quantified by Image J software (version 1.48). (**D**,**E**) Total PA content (**D**) and PA species (**E**) in the siliques. PA was extracted from siliques 30 days after pollination. PA was separated in TLC and the PA level was quantified by gas chromatography. Values are mean ± SD (*n* = 3). * and ** denote significance at *p* < 0.05 and *p* < 0.01, respectively, compared with WT based on Student’s *t*-test.

GPAT catalyzes the acylation of G3P to produce LPA, which was subsequently acylated to generate PA, an important intermediate in lipid metabolism. To test the effect on PA production, BnaGPAT9 was expressed in *E. coli* cells, and PA product was analyzed after separation by thin layer chromatography (TLC, Figure 6A,B). PA level in *E. coli* cells harboring BnaGPAT9 was much higher than in the control cells containing empty vector pET42a (Figure 6C). To further test the effect of BnaGPAT9 on PA biosynthesis in vivo, PA level in siliques was analyzed. The results showed that PA levels in the siliques of *BnaGPAT9*-OE27, OE33, and OE80 lines were significantly higher than that of WT, increased by 36.2%, 43.5%, and 55.2%, respectively (Figure 6D). Increased PA in *BnaGPAT9*-OE siliques resulted from increased 18:1 and 18:2 fatty acids (Figure 6E). These results suggest that increased expression of BnaGPAT9 promotes PA production in vitro and in vivo.

### 2.5. BnaGPAT9 Affected Membrane Phospholipids and Enhanced Eukaryotic Galactolipids in Developing Seed

**Figure 7:**
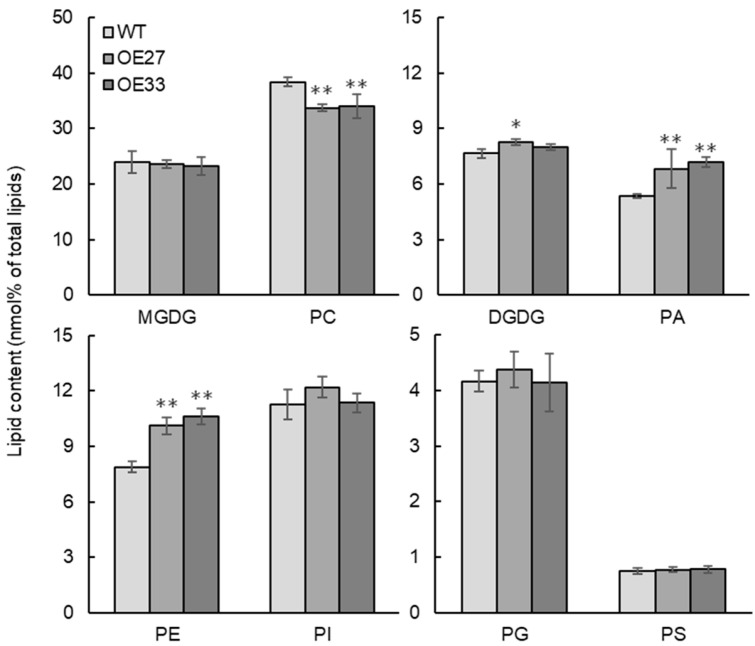
Overexpression of *BnaGPAT9* altered phospholipids and galactolipids in developing seeds. Lipids were extracted from developing seeds 30 days after pollination. Values are mean ± SD (*n* = 4). * and ** denote significance at *p* < 0.05 and *p* < 0.01, respectively, compared with WT based on Student’s *t*-test. PA, phosphatidic acid; PC, phosphatidylcholine; PE, phosphatidylethanolamine; PI, phosphatidylinositol; PG, phosphatidylglycerol; PS, phosphatidylserine; MGDG, monogalactosyldiacyglycerol; DGDG, digalactosyldiacylglycerol.

**Figure 8:**
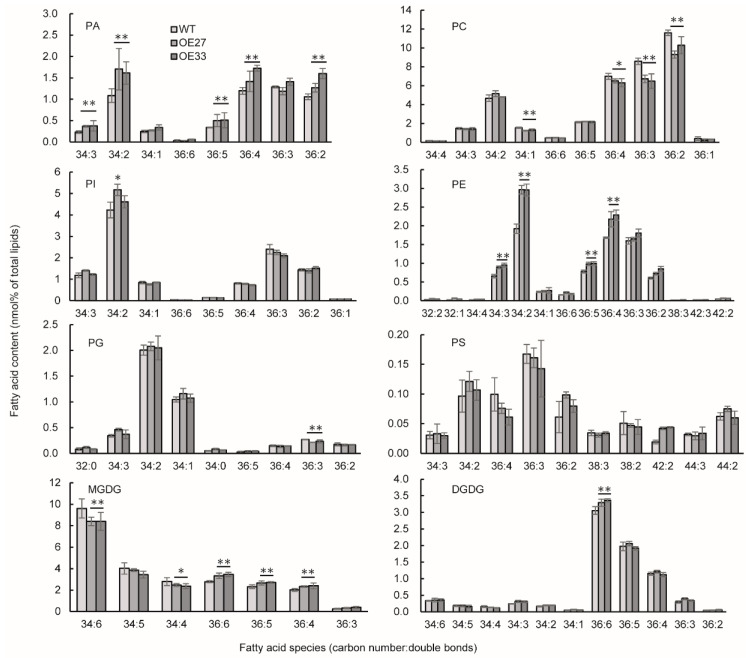
Overexpression of *BnaGPAT9* altered membrane lipid composition in developing seeds. Lipids were extracted from developing seeds 30 days after pollination. Fatty acid species were shown as total acyl carbons: total double bonds. Values are mean ± SD (*n* = 4). * and ** denote significance at *p* < 0.05 and *p* < 0.01, respectively, compared with WT based on Student’s *t*-test.

In *Brassica napus*, the developing seed not only serves as an oil storage organ but also plays an important role in photosynthesis to provide carbon source for embryonic development and growth. To investigate how BnaGPAT9 affects oil accumulation, we selected *BnaGPAT9*-OE27 and OE33 as the representative lines for lipid profiling in developing seeds 30 days after pollination (DAP) by electron-spray ionization tandem mass spectrometry (ESI-MS/MS). The results showed that PA contents in 30-DAP seeds of *BnaGPAT9*-OE27 and OE33 were significantly higher than that of WT, increased by 27.4% and 33.8%, respectively (Figure 7), which resulted from increased 34:2-, 34:3-, 36:2-, 36:4- and 36:5-PA (Figure 8).

In developing seeds, phospholipid PC has dual roles, functioning as a structural component of cellular membranes and a precursor for TAG biosynthesis. Lipid profiling showed that PC is a major phospholipid in developing seeds of WT, which consists of 38.6% total polar lipids tested (Figure 7). PC in *BnaGPAT9*-OE developing seeds was significantly lower than that of WT, decreased by 12.2% and 11.5% in OE27 and OE33 seeds, respectively (Figure 7). The most abundant PC species include 34:2-, 36:2-, 36:3-, and 36:4-PC, and decreased PC in *BnaGPAT9*-OE seeds resulted from the reduction in 34:1-, 36:2-, 36:3-, and 36:4-PC species (Figure 8). In addition, the lysoPC content in *BnaGPAT9*-OE developing seeds was also significantly lower than that of WT due to reduced 18:1-LysoPC (Supplementary Figure S2). Conversely, PE content in *BnaGPAT9*-OE seeds was substantially higher than that of WT (Figure 7). The increased PE was attributed to increased 34:2-, 34:3-, 36:4-, and 36:5-PE species (Figure 8). By comparison, the contents of PG, PI, and PS in *BnaGPAT9*-OE seeds were not significantly different from that of WT (Figure 7 and Figure 8). Together, the results suggest that overexpression of *BnaGPAT9* led to a substantial alteration of phospholipids with decreased PC and lysoPC and increased PE in developing seeds.

Moreover, the galactolipid DGDG content in *BnaGPAT9*-OE seeds was also higher than that of WT (Figure 7) due to increased 36:6-DGDG (Figure 8). Although a total content of MGDG was less affected by BnaGPAT9, overexpression of *BnaGPAT9* led to increased 36:4-, 36:5-, and 36:6-MGDG and a corresponding decrease in 34:4- and 34:6-MGDG (Figure 8). These results suggest that BnaGPAT9 affects the content and composition of photosynthetic membranes via enhancing the eukaryotic pathway and reducing the prokaryotic pathway of galactolipid synthesis.

## 3. Discussion

Phospholipids and storage lipid TAG are mainly synthesized via the eukaryotic pathway in the ER [8,23]. The eukaryotic glycerolipids also serve as precursors for chloroplast membrane lipids in plants [17,18,19,20]. The first acylation at the sn-1 position of G3P catalyzed by GPAT is a committed step for the production of TAG and membrane glycerolipids in various organisms [8]. Of nine GPATs involved in the eukaryotic pathway in *Arabidopsis*, eight GPATs, GPAT1–GPAT8, are involved in the synthesis of extracellular lipids such as cutin and suberin rather than intracellular lipids [27,28,30,34,35,36]. Recently, GPAT9 has been identified to be responsible for TAG synthesis and is probably involved in phospholipids synthesis in *Arabidopsis* [15,16]. However, the gene encoding GPAT that is responsible for oil synthesis remains to be elucidated in *Brassica napus.* Furthermore, the role of plant GPAT9 in the biosynthesis of phospholipids and galactolipids is less understood due to the lethality of male and female gametophytes resulting from loss of GPAT9 in *Arabidopsis*. Here, we showed that overexpression of *BnaGPAT9* promoted oil accumulation and enhanced eukaryotic galactolipid biosynthesis in developing seeds, which provides molecular genetic evidence for the improvement of seed oil content in *Brassica napus*.

GPAT9 catalyzes the first acylation of G3P to produce LPA that is rapidly acylated at the sn-2 position to produce PA. It has been shown that *Arabidopsis* GPAT9 played an important role in TAG accumulation [15,16], but its effect on PA production is not clear. PA is an important intermediate in lipid metabolism, which provides a precursor for both TAG and membrane lipids [7,8]. To determine how GPAT9 affects lipid flux into TAG, PA and its molecular species were analyzed in vitro and in vivo, and were compared to those molecular species of TAG in developing seeds in this study. Our results from in BnaGPAT9 expressed in *E. coli* cells and *Brassica napus* plants showed that overexpression of *BnGPAT9* increased PA production, and increased PA is rich in 18:2-PA species such as 34:2 (16:0/18:2)-PA and 36:4 (18:2/18:2)-PA, which is similar to elevated 18:2-containing TAG in *BnaGPAT9*-OE seeds, and elevated 34:2- and 36:4-PE as well. Our results suggest that BnaGPAT9 promotes PA production, thereby enhancing PA flux to TAG synthesis. In vegetative tissues, PA is a minor phospholipid, consisting of 1–4% of total polar lipids [7]. It has been shown that PA is highly accumulated in reproductive organs such as flowers [37]. Our results also showed a higher PA level in developing seeds (Figure 7), as compared to that of leaves [38]. The results suggest that high PA promotes oil synthesis in embryo in *Brassica napu*. PA functions as not only a lipid intermediate but also a signal molecular in diverse biological processes [7,39]. How PA affects oil accumulation needs to be explored further in future studies.

*Arabidopsis* GPAT9 contributed to total phospholipids [16]. However, the effect of GPAT9 on individual phospholipids remains to be elucidated in plants, particularly oil crop plants. Here, we showed that overexpression of *BnaGPAT9* led to increased oil content accompanied by reduced PC level with decreased 36C-PC species, suggesting decreased 36C-PC (two 18C acyl groups at PC) flux into TAG in *BnaGPAT9*-OE seeds. PC is a major site for fatty acid desaturation in plants [23,40]. It has been suggested that acyl group derived from PC into TAG has multiple points including de novo synthesis of glycerolipids by GPAT9/LPAT2/LCAT2 or exchange between PC and DAG by PDCT/DGAT1 [15,41,42]. PC-edited acyl group is a major source of acyl-CoA for de novo glycerolipid synthesis in Kennedy pathway enzymes such as GPAT, LPAT, and DGAT [41,43,44,45,46]. It has been shown that GPAT9 interacts with AtLPCAT2 in *Arabidopsis* [15]. Our results showed that *BnaGPAT9*-OE increased 18:2-containing TAG, accompanied by reduced 18C containing PC (36C-PC) species. These results imply that GPAT9 may primarily use PC-derived 18C-acyl group for TAG synthesis. Further study is needed to investigate BnaGPAT9 involved in linkage between PC and TAG in developing seeds. In contrast, our lipid profiling data showed that the PE content in *BnaGPAT9*-OE seeds had a significant increase relative to WT. In addition to a precursor for TAG synthesis, PC also serves as a structural component of cellular membranes [8,40,41,42]. Increased PE in *BnaGPAT9*-OE seeds may be due to a complementary effect of reduced PC, thereby governing lipid homeostasis to maintain cellular membrane structural integration. By comparison, other phospholipids such as PG and PS were less affected by BnaGPAT9. It has been shown that 85% PG in plastid is derived from the prokaryotic pathway [47]. *Arabidopsis ats1* mutant exhibited a defective prokaryotic PG [12]. Our previous results from plastidial *BnaATS1*-OE plants displayed increased PG content [38]. However, overexpression of this ER-localized BnaGPAT9 had little effect on PG content and composition. These results suggest that BnaGPAT9 is involved in the eukaryotic pathway, predominantly affecting PC and PE metabolism during seed developing to enhance seed oil synthesis in *Brassica napus*.

Galactolipids such as MGDG and DGDG can be derived from both eukaryotic and prokaryotic pathways in many plants [8,17,18,19,20]. The fatty acid species of galactolipids derived from two pathways are usually distinct at the sn-2 position of glycerol backbone. MGDG derived from the eukaryotic pathway contains predominantly a C18 fatty acid at the sn-2 position of glycerol backbone, whereas that of the prokaryotic pathway possesses a C16 fatty acid at the same position [8]. Loss of plastidial GPAT (ATS1) resuls in defective prokaryotic thylakoid lipids, and the thylakoid membranes in the *ats1* mutant are mainly synthesized through the eukaryotic pathway [12,48]. By contrast, *Arabidopsis* plants with defective eukaryotic lipid transport exhibit a substantial reduction in eukaryotic thylakoid lipids [17,18]. Whether GPAT9 affects photosynthetic membrane lipids is unclear. Here, we showed that ER-localized BnaGPAT9 promoted DGDG production with elevated 36:6-DGDG species. Furthermore, overexpression of *BnaGPAT9* also led to increased 36:6 (18:3/18:3)-MGDG and decreased 34:6 (18:3/16:3)-MGDG. These results suggest that BnaGPAT9 promotes a substantial shift from the prokaryotic pathway (16:3) toward the eukaryotic pathway (18:3) of galactolipid synthesis. This alteration of membrane lipids caused by *BnaGPAT9* overexpression is somehow similar to the results of the *ats1* mutant, a mutagenesis of prokaryotic *GPAT* (*ATS1*) in *Arabidopsis* [12,48]. Our previous study showed that plastid-localized *BnaATS1*-OE plants exhibited an elevation of prokaryotic 34:6- and 34:5-MGDG [40]. Together, these results suggest that overexpression of *BnaGPAT9* enhances eukaryotic galactolipid synthesis in *Brassica napus*.

## 4. Materials and Methods

### 4.1. Plant Materials and Growth Conditions

The rapeseed cultivar Westar (*Brassica napus*) was used as wild-type (WT) control and plants of *BnaGPAT9* overexpression were generated in Westar genetic background in this study. Seeds were sown in pots containing soil and two-week-old seedlings were transplanted to pots containing soil in growth room under the conditions of 14 h light (25 °C)/10 h dark (20 °C), with light density of 200–300 mmol.m^−2^.s^−1^ and relative humidity of 60%. The plants were also grown in pots or in the fields with regular watering under natural conditions from autumn through the spring seasons in Wuhan, China.

### 4.2. Database Search and Sequence Analysis

The amino acid sequences of *Arabidopsis* GPATs were obtained from the *Arabidopsis* Information Resource (http://www.arabidopsis.org/, accessed on 20 November 2018) using key words of glycerol-3-phosphate acyltransferase or conserved domain of acyltransferase. The *Brassica napus* GPAT members were identified by BLAST search in the *Brassica napus* databases querying with the *Arabidopsis* GPAT sequences combined with using keywords of glycerol-3-phosphate acyltransferase. The amino acid sequences of GPATs in *Brassica napus* were obtained from *Brassica napus* pan-genome information resource (http://cbi.hzau.edu.cn/bnapus/index.php, accessed on 20 November 2018), *Brassica napus* Genome Browser (https://www.genoscope.cns.fr/brassicanapus/, accessed on 20 November 2018), and National Center for Biotechnology Information (http://www.ncbi.nlm.nih.gov, accessed on 20 November 2018). Amino acid sequence alignment was analyzed by using MAFFT software (version 7.490). The phylogenetic analysis was constructed by using Mega 11 software (version 11.0.13), with the Neighbor-Joining method, Poisson model, and 1000 Bootstrap tests. The phylogenetic analysis was embellished by EvolView. The domain and motif were retrieved from InterPro database (https://www.ebi.ac.uk/interpro/, accessed on 20 November 2018).

### 4.3. BnaGPAT9 Cloning, Vector Construction, and Plant Transformation

Total RNA was isolated from leaves using the TransZol reagent (TransGen Biotech, Beijing, China) and digested with DNase I to remove genomic DNA. The resultant RNA was used for synthesis of first-strand cDNA by reverse transcription using a TransScript cDNA Synthesis SuperMix Kit (TransGen Biotech, Beijing, China). The full length of the *BnaC01GPAT9* (BnaC01T0014600WE) coding sequence (CDS) was amplified from cDNA by PCR using forward primer 5′-TCTAGAATGAGCAGCACGGCAGGAA-3′ paired with reverse primer 5′-GGATCCTCACTTGTCTTCCAATCTAGC-3′. The purified *BnaGPAT9* CDS was ligated into binary vector pBI121 after digestion by *Xba*I and *Bam*HI under the control of 35S promoter. The resultant construct was transformed into *Agrobacterium tumefaciens* GV3101 strain for infection in hypocotyls of Westar to obtain regeneration plants according to a protocol described previously [49]. The transgenic plants were identified by PCR using the primer specific to the pBI121 vector sequence 5′-GATGGTTAGAGAGGCTTACGCA-3′ paired with *BnaC01GPAT9* (BnaC01T0014600WE)-specific primer *BnaGPAT9*-OE-R 5′-GGATCCTCACTTGTCTTCCAATCTAGC-3′ (Supplementary Table S1).

### 4.4. Quantitative Reverse-Transcription PCR

Total RNA was extracted from various tissues of cv. Westar plants using Transzol reagent (TransGen Biotech, Beijing, China). After genomic DNA was removed by DNase I digestion, the resultant RNA was used as a template to synthesize the first-strand cDNA by reverse-transcription PCR (RT-PCR) using a TransScript cDNA Synthesis SuperMix Kit according to the manufacturer’s instructions (TransGen Biotech, Beijing, China). cDNA with similar concentration was used for quantitative RT-PCR (RT-qPCR) using *BnaActin* (BnaC02T0031300WE) as an internal standard. RT-qPCR was performed with a MyIQ real-time PCR system (Bio-Rad, http://www.bio-rad.com, accessed on 20 October 2023) using TransStart Green qPCR SuperMix (TransGen Biotech, Beijing, China) under conditions of 95 °C for 30 s, 55 °C for 30 s, and 72 °C for 30 s, for 55 cycles. All primers used are listed in Supplementary Table S1.

### 4.5. Subcellular Localization

The full-length *BnaC01GPAT9* (BnaC01T0014600WE) CDS was amplified from cDNA by PCR using primers 5′- GAGCTCATGAGCAGCACGGCAGGAA-3′ and 5′-GGATCCCTTCTCTTCCAATCTAGCCA-3′. The purified PCR product was ligated into the pCAMBIA1301s vector in-frame with C-terminal fusion to GFP. The *Agrobacterium* GV3101 strain harboring the construct and the ER localized marker CD3-953 were infiltrated into the leaves of four-week-old *Nicotiana benthamiana*. Four days after infection, the fluorescent image was observed using a confocal laser scanning microscope (Leica, Biberach, Germany).

### 4.6. GPAT Activity Assay

The full-length *BnaC01GPAT9* (BnaC01T0014600WE) CDS was amplified from cDNA by PCR using primers 5′-TCTAGAATGAGCAGCACGGCAGGAA-3′ and 5′- GGATCCCTTGTCTTCCAATCTAGC-3′. The purified PCR product was ligated into the pET42a vector after digestion with *Sac*I and *Bam*HI. The construct was transformed into *E. coli* BL21 cells for protein expression. The BnaGPAT9 activity was assayed in 100 μL of reaction mixtures containing 500 μM G3P-Ca, 80 μM 18:1-CoA, 50 mM Tris·HCl (pH7.5), 2 mM MgCl_2_, 1 mM DTT, and 100 μg total proteins. The reaction mixtures were incubated at 30 °C for 50 min and stopped by the addition of 300 μL CHCl_3_:CH_3_OH (2:1). The resultant lipids of the reaction were loaded onto a thin-layer chromatography (TLC) plate, developed with CHCl_3_: CH_3_CH_2_OH: Et3N: H_2_O (10:11.3:11.7:2.7, *v*/*v*). PA in TLC was visualized by solution (0.04 g bromothymol blue in 100 mL 0.01 M NaOH). The relative PA level was quantified by Image J software (version 1.48).

### 4.7. Lipid Extraction and Analysis

The developing seeds were sampled from siliques 30 days after pollination and immersed in 75 °C isopropanol containing 0.02% butylated hydroxytoluene (BHT) for 15 min. After being cooled to room temperature, chloroform and water were added and incubated for 1 h by gentle shaking. The extracts were transferred to clean glass tubes, and the remaining samples were re-extracted with chloroform: methanol (2:1, *v*/*v*) until the leaves became bleached. The extracts from the same sample were combined and then washed with 1 M KCl, followed by another wash with water. The resultant extracts were dried under a stream of nitrogen, and then dissolved in a defined volume of chloroform. Lipids were quantitatively profiled by electron-spray ionization tandem mass spectrometry (ESI-MS/MS) as described previously [50] using internal standards including di14:0-PG, diPhy (20:0)-PG, di12:0-PE, diPhy (20:0)-PE, di23:0-PE, 13:0-lysoPC, 19:0-lysoPC, di12:0-PC, di24:1-PC, di14:0-PA, diPhy (20:0)-PA, di14:0-PS, diPhy-PS, 16:0–18:0-PI, di18:0-PI, 34:0-DGDG, 36:0-DGDG, 34:0-MGDG, and 36:0-MGDG. The seed oil content was measured by gas chromatography (GC) (Agilent 7890A, Santa Clara, CA, USA) based on a previous method [49].

## Data Availability

Data are contained within the article and supplementary materials.

## Supplementary Materials

The following supporting information can be downloaded at: https://www.mdpi.com/article/10.3390/ijms242216111/s1.
