# Supplementary material for: Glycerol-3-Phosphate Acyltransferase GPAT9 Enhanced Seed Oil Accumulation and Eukaryotic Galactolipid Synthesis in Brassica napus
Source: Int J Mol Sci. 2023 Nov 9;24(22):16111. doi: 10.3390/ijms242216111 (PMC10671787; doi:10.3390/ijms242216111)
Supplement: Supplementary file 1 [file ijms-24-16111-s001.zip › ijms-2647118-supplementary.pdf]

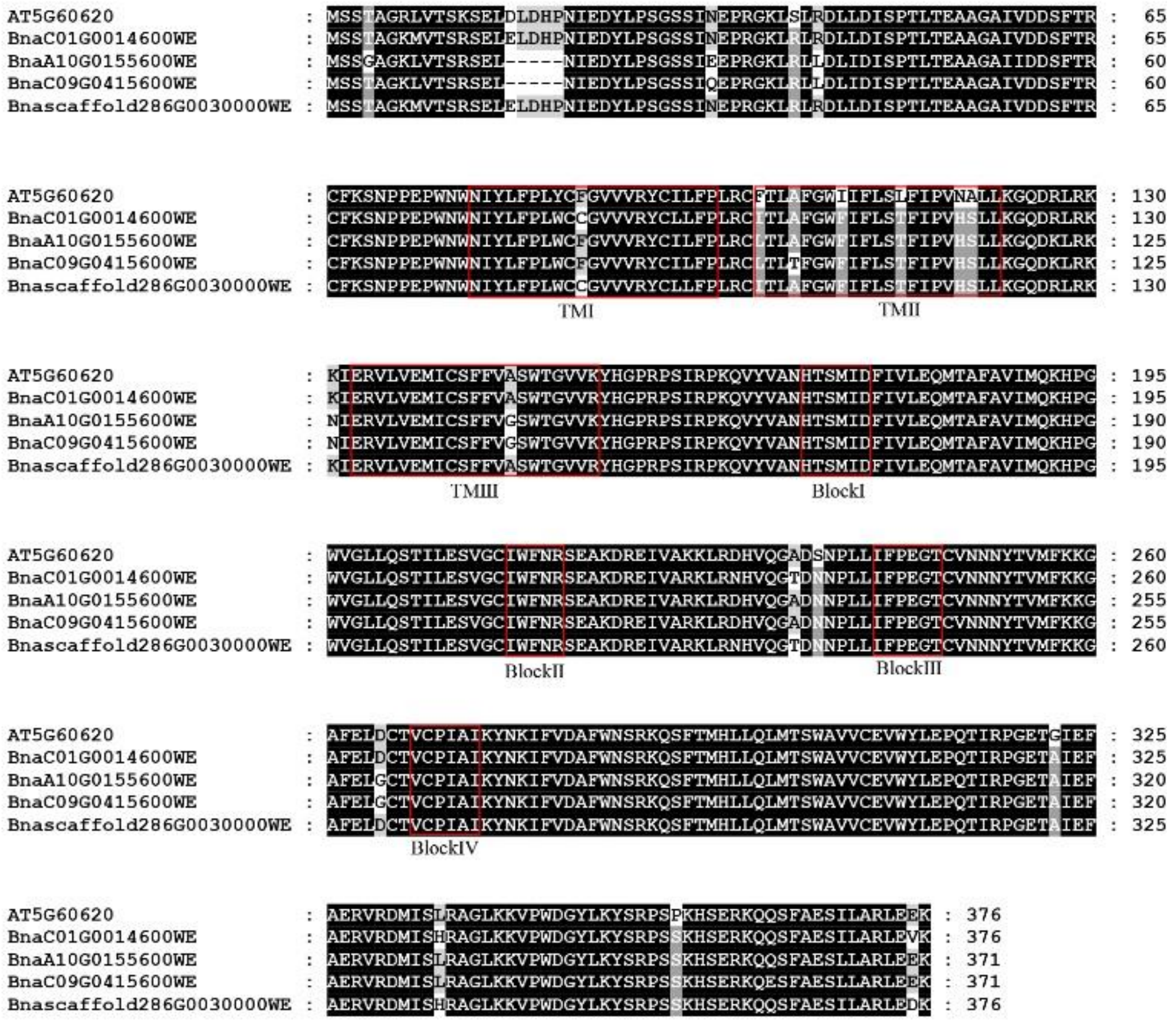


**Supplementary Figure S1**. Sequence alignment of GPATs from different plants. Amino acid sequences were compared by MATTF software and colored using the GeneDoc software. The identical and similar residues are shaded in black and grey, respectively. TM I-III, the transmembrane domain I, II and III. BlockI-IV, the motifs conserved in catalytic domain of acyltransferase. At, *Arabidopsis thaliana*; Bna, *Brassica napus*.

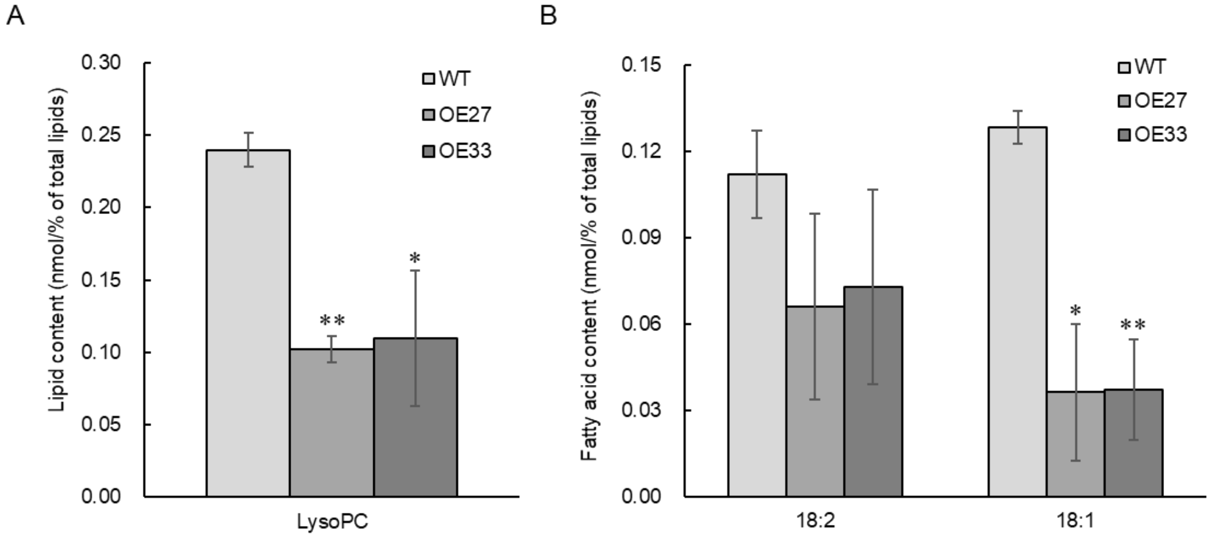


**Supplementary Figure S2.** Overexpression of *BnaGPAT9* led to reduced lysoPC in developing seeds. (**A**) Total lysoPC in developing seeds. (**B**) Fatty acid species of lysoPC in developing seeds. Lipids were extracted from developing seeds of 30 days after pollination. Fatty acid species were shown as total acyl carbons: total double bonds. LysoPC, lysophosphatidylcholine. Values are mean ± SD (*n* = 3). * and ** denote significance at P < 0.05 and P < 0.01, respectively, compared with WT based on Student's *t* test.

**Supplementary Table S1.** Primers used in this study.

| Primer names | Primer sequences (5'→3') | Purposes |
|---|---|---|
| *BnaGPAT9*-OE-F | TCTAGAATGAGCAGCACGGCAGGAA | Overexpression |
| *BnaGPAT9*-OE-R | GGATCCTCACTTGTCTTCCAATCTAGC | |
| *BnaGPAT9*-PF | TCTAGAATGAGCAGCACGGCAGGAA | Protein expression |
| *BnaGPAT9*-PR | GGATCCCTTGTCTTCCAATCTAGC | |
| *BnaGPAT9*-GFP-F | GAGCTCATGAGCAGCACGGCAGGAA | Localization |
| *BnaGPAT9*-GFP-R | GGATCCCTTCTCTTCCAATCTAGCCA | |
| RT-*BnaActin*-F | TGTTCCCTGGAATTGCTGACCGTA | RT-qPCR |
| RT-*BnaActin*-R | TGCGACCACCTTGATCTTCATGCT | |
| RT- *BnaGPAT9*-F | TGTTGACGCCTTCTGGAATAG | |
| RT- *BnaGPAT9*-R | TCCAAGTACCACACTTCACATAC | |
| BnaC01T0014600WE-F | TTCACCCGGCGATCGGAAAGTGTC | |
| BnaC01T0014600WE-R | GGCACCAGCAGCTTCAGTC | |
| BnaC09T0415600WE-F | CACCACAACCAATTCACCCGGC | |
| BnaC09T0415600WE-R | TCTCGTGAATGAGTCATCAAT | |
| BnaA10T0155600W-F | CGGAAGCGAGAGAGAGAGAGAG | |
| BnaA10T0155600W-R | GCTCTGGAGGGTTCGACTTA | |
| BnascaffoldWE-F | TCGGAAGAGAGAGAGAGAGAGATG | |
| BnascaffoldWE-R | GCTCTGGAGGGTTCGACTTG | |
